# Different specificities of two aldehyde dehydrogenases from *Saccharomyces cerevisiae var. boulardii*

**DOI:** 10.1042/BSR20160529

**Published:** 2017-03-02

**Authors:** Suprama Datta, Uday S. Annapure, David J. Timson

**Affiliations:** 1 Department of Food Engineering and Technology, Institute of Chemical Technology (ICT), Matunga, Mumbai 400 019, India; 2 School of Biological Sciences, Queen’s University Belfast, Medical Biology Centre, 97 Lisburn Road, Belfast BT9 7BL, U.K.; 3 School of Pharmacy and Biomolecular Sciences, University of Brighton, Huxley Building, Lewes Road, Brighton BN2 4GJ, U.K.

**Keywords:** aliphatic aldehyde, aromatic aldehyde, cooperative kinetics, enzyme specificity, tetrameric protein, Yeast aldehyde dehydrogenase

## Abstract

Aldehyde dehydrogenases play crucial roles in the detoxification of exogenous and endogenous aldehydes by catalysing their oxidation to carboxylic acid counterparts. The present study reports characterization of two such isoenzymes from the yeast *Saccharomyces cerevisiae var. boulardii* (NCYC 3264), one mitochondrial (Ald4p) and one cytosolic (Ald6p). Both Ald4p and Ald6p were oligomeric in solution and demonstrated positive kinetic cooperativity towards aldehyde substrates. Wild-type Ald6p showed activity only with aliphatic aldehydes. Ald4p, on the contrary, showed activity with benzaldehyde along with a limited range of aliphatic aldehydes. Inspection of modelled structure of Ald6p revealed that a bulky amino acid residue (Met^177^, compared with the equivalent residue Leu^196^ in Ald4p) might cause steric hindrance of cyclic substrates. Therefore, we hypothesized that specificities of the two isoenzymes towards aldehyde substrates were partly driven by steric hindrance in the active site. A variant of wild-type Ald6p with the Met^177^ residue replaced by a valine was also characterized to address to the hypothesis. It showed an increased specificity range and a gain of activity towards cyclohexanecarboxaldehyde. It also demonstrated an increased thermal stability when compared with both the wild-types. These data suggest that steric bulk in the active site of yeast aldehyde dehydrogenases is partially responsible for controlling specificity.

## Introduction

The multigene family of aldehyde dehydrogenases (EC 1.2.1.3) contributes primarily to acetaldehyde detoxification through its oxidation to acetate [1]. Other physiological roles of aldehyde dehydrogenases include lipid peroxidation [2], the metabolism of amino acids and biogenic amines [3,4], corticosteroids [5], retinoids [6,7] and protein deglycation [8]. The superfamily also participate in detoxification of exogenous aldehydes from beverages, foods, industrial pollutants etc [9,10]. Mammalian aldehyde dehydrogenases are classified on the basis of kinetic mechanistic features into three classes of enzyme family: ALDH1, ALDH2 and ALDH3 being the representative members with rate-limiting steps in NADH dissociation, deacylation and hydride transfer respectively [11,12]. All three classes conform to an ordered ternary complex kinetic mechanism in which the NAD(P)^+^ coenzyme binds first in the reaction sequence [13].

ALDH superfamily members typically exhibit a broad substrate specificity and many of them are able to oxidize several highly reactive aliphatic and aromatic aldehydes. Exogenous aldehydes, whether intermediates or products, may be derived from the metabolism of drugs (e.g. ethanol, cyclophosphamide and ifosfamide) or substances present in the environment (i.e. smog, cigarette smoke and vehicle exhaust fumes) or may even be introduced as such [9,14–17]. Endogenous aldehydes derive from the metabolism of amino acids, biogenic amines, vitamins, steroids or lipids. Therefore, degradation of exogenous and endogenous aldehydes is of utmost importance [9,10,18]. Hence, there is a need to understand the biochemistry of a diverse range of aldehyde dehydrogenases.

*Saccharomyces cerevisiae* (budding yeast) aldehyde dehydrogenases have been classified into cytosolic and mitochondrial enzymes. *ALD2 *(*YMR170C*),* ALD3 *(*YMR169C*),* ALD4 *(*YOR374W*),* ALD5 *(*YER073W*) and *ALD6 *(*YPL061W*) are the five different gene products identified in these categories [19,20]. Two cytosolic (Ald2p and Ald3p) and one mitochondrial (Ald5p) isoenzymes are the best studied ones. The constitutively expressed, K^+^-activated Ald5p is reported to be involved in regulation or biosynthesis of electron transport chain components and acetate formation [20–22]. Stress-induced expression of the cytosolic Ald2p and Ald3p is required for ethanol oxidation and β-alanine synthesis [23]. Cytosolic Ald6p (Mg^2+^ activated) and mitochondrial Ald4p are less well studied, and are reported to be involved in anaerobic growth and conversion of acetaldehyde into acetate [21,22,24,25]. Wang et al. documented the substrate specificity of *S. cerevisiae* mitochondrial Ald4p (previously known as ALDH2) along with human ALDH1 and ALDH2 towards various aliphatic and aromatic aldehydes [26]. The enzyme was demonstrated to be active with a range of aliphatic aldehydes and a small number of cyclic or aromatic ones [26].

In the present study, we investigated the specificity of two yeast aldehyde dehydrogenases for linear/branched-chain aliphatic aldehydes and cyclic/aromatic aldehydes as substrates. The genes encoding the mitochondrial and cytosolic isoenzymes *ALD4* and *ALD6* were cloned from a fruit isolate of *Saccharomyces cerevisiae* *var. boulardii*, NCYC 3264 (formerly known as *Saccharomyces boulardii*) [27]. This yeast strain has attracted particular attention as a probiotic and also for accelerating wound healing, although conclusive evidence of its effectiveness in these applications is limited [28–31]. The strain produces a variety of molecules with antioxidant properties, including some aldehydes and their oxidation products [32]. Given that genes encoding enzymes of the polypropanoid pathway appear to be absent from the genome of *S. cerevisiae* *var*. *boulardii*, we reasoned that aldehyde dehydrogenases may be important in the production of these compounds providing a further justification for studying their biochemical properties. Recombinant Ald4p and Ald6p proteins from *S. cerevisiae* *var*. *boulardii* were expressed for functional characterization. Based on molecular models of the proteins, a hypothesis to explain the different substrate specificities was advanced. A variant of Ald6p was also expressed to test this hypothesis.

## Materials and methods

### *S. cerevisiae* strains and growth conditions

*S. cerevisiae var. boulardii* NCYC 3264 strain was procured from National Collection of Yeast Cultures (Norwich, U.K.). Cultures were grown in YEPD (1% yeast extract, 2% peptone and 2% glucose) medium at 30°C.

### Recombinant expression and purification of Ald6p and Ald4p

Genomic DNA was extracted from yeast cells using the YeaStar genomic DNA kit ™ (Zymo Research Corp., U.S.A.). The coding sequences for *ALD6* and *ALD4* were PCR-amplified from this NCYC 3264 genomic DNA. The primers were designed based on the ORF of *Saccharomyces sp. ‘boulardii’* whole genome shotgun sequence (available at: http://www.ncbi.nlm.nih.gov/nuccore/?term=saccharomyces%20boulardii%20genome%20) [33]. The amplicons were inserted into the *Escherichia coli* expression vector pET46 Ek/LIC (Merck-Millipore, Nottingham, U.K.) according to the manufacturer’s instructions (note that this vector introduces bases coding for the amino acid sequence MAHHHHHHVDDDDK at the 5’-end of the coding sequence). Correct insertion into the vector was verified by PCR and by DNA sequencing (GATC, London, U.K.) of the insert.

The expression vector was used to transform competent *E. coli* Rosetta™ (DE3) cells (Merck-Millipore) and colonies resulting from this transformation were used to inoculate cultures (5 ml of LB medium supplemented with 100 µg·ml^−1^ ampicillin and 34 µg·ml^−1^ chloramphenicol), which were grown overnight at 37°C with shaking. Each culture was then diluted into 1 litre of LB (supplemented with 100 µg· ml^−1^ ampicillin and 34 µg·ml^−1^ chloramphenicol), grown until *A*_600_ reached 0.6 to 1.0 (typically 5–6h) at 30°C, followed by a slow induction by adding 1.3 mM IPTG overnight (12–16 h) at 16°C. Cells were harvested by centrifugation (4200***g*** for 15 min at room temperature), resuspended in cell resuspension buffer (50 mM HEPES–OH, pH 7.5, 150 mM NaCl, 10% (v/v) glycerol) and stored frozen at −80°C until the purification step.

For purification, cell suspensions were thawed, disrupted by sonication on ice (three pulses at 100 W for 30 s with 30 s gaps for cooling) and clarified by centrifugation (20000***g*** for 20 min at 4°C). The supernatant was applied to a cobalt agarose column (1 ml, His-Select, Sigma, Poole, U.K.), which had been pre-equilibrated in buffer A (cell resuspension buffer, except 500 mM NaCl) and allowed to pass through by gravity. The column was washed twice with 40 ml of buffer A and the protein was eluted with two 2 ml aliquots of buffer C (buffer A plus 250 mM imidazole). Protein containing fractions were identified by SDS/PAGE and dialysed overnight at 4°C against cell resuspension buffer supplemented with 1 mM DTT. The concentration of Ald6p and Ald4p were determined by the method of Bradford [34] using BSA as a standard. The purified fractions were frozen at −80°C in 20 μl aliquots.

A mutation resulting in a change to the coding sequence of *ALD6* (p.M177V) was generated by site-directed mutagenesis using the QuikChange method [35]. The altered plasmid sequence was verified by Sanger sequencing (GATC, London, U.K.) and transformed into *E. coli* Rosetta™(DE3) (Merck-Millipore) competent cells. Expression and purification were carried out using the same protocols as above.

### Bioinformatics and molecular modelling

The well-studied human retinal dehydrogenase 1 [accession no: NP_000680.2] (class1) and liver mitochondrial ALDH [accession no: CAG33272.1] (class 2) were identified using protein BLAST search. Multiple sequences of the above mentioned ALDHs along with Ald4p and Ald6p sequences from the present study were aligned using Transitive Consistency Score (TCS) web server available from http://tcoffee.crg.cat/tcs to determine their homology and conservation of domain residues [36]. The sequence homology was evaluated using ESPript 3.0 available at http://espript.ibcp.fr [37]. A BLOSUM50 matrix scoring the similarity and identity between the sequences was computed using the MatGAT v2.01 application [38].

The predicted protein sequences of Ald4p and Ald6p were submitted to Phyre2 in the intensive mode to generate an initial molecular monomeric model of the protein [39]. This model was then minimized and computationally solvated using YASARA (http://www.yasara.org/minimizationserver.htm) [40]. To generate a tetrameric model, four copies of the model were aligned (using PyMol) to the four subunits of sheep class 1 ALDH (PDB ID: 1BXS [41]), which shares >90% protein sequence similarity with human retinal dehydrogenases ALDH1A1 (PDB ID: 4WB9 [42]) as judged by the Sequence Similarity Cutoff parameters in Protein Data Bank (PDB). The tetrameric structure was saved in a new pdb file together with the NAD^+^ cofactors associated with each subunit. This tetrameric, cofactor bound structure was then minimized using YASARA. To identify the aldehyde-binding site, the structure of human mitochondrial aldehyde dehydrogenase complexed with crotonaldehyde was used (PDB: 1O01 [43]) and was aligned to the tetrameric models of Ald4p and Ald6p. A new pdb file was generated for both Ald4p and Ald6p incorporating crotanaldehyde, which was then minimized in YASARA. Subsequent models were generated with other aldehydes (octanal and benzaldehyde) bound by overlaying the ‘new’ substrate over the aldehyde group and key atoms from the carbon backbone, removing the ‘old’ substrate and reminimizing the complex in YASARA. These models are provided as supplementary information.

### Cross-linking

Cross-linking with bis(sulfosuccinimidyl) suberate (BS^3^, 50–800 µM) was carried out with 15 µM protein (diluted as required in 100 mM sodium phosphate buffer, pH 7.4) in a total volume of 10 µl. Reaction mixtures were incubated at 30°C for 30 min before addition of the cross-linker and then incubated at the same temperature for a further 35 min. Reactions were stopped by addition of an equal volume of SDS-loading buffer (120 mM Tris/HCl, pH 6.8, 4% (w/v) SDS, 20%(v/v) glycerol, 5% (w/v) Bromophenol Blue and 1% (w/v) DTT) and analysed by SDS/PAGE (10% gel).

### Analytical gel filtration

Ald6p (wild-type and p.M177V variant) and Ald4p (200 µl of a 60 µM purified protein aliquot) were chromatographed on a Sephacryl S-300 (Pharmacia) column (total volume, *V*_t_ = 65.2 ml; void volume, *V*_0_ = 15.1 ml) at a flow rate of 1 ml·min^−1^. The column was equilibrated and developed in buffer G (50 mM Tris/HCl, 17 mM Tris-base, 150 mM sodium chloride, pH 7.4) [44–46]. Fractions (1 ml) were collected and analysed for protein content by measuring the absorbance at 280 nm. Standard proteins (Thyroglobulin, 669 kDa; Albumin, 67 kDa; Chymotrypsinogen, 25 kDa and Ribonuclease A, 14 kDa) were used to calibrate the column. Their elution volumes (*V*_e_) were used to calculate *K*_av_ according to the equation:

$$K_{\text{av}}=(V_{e}-V_{0})/(V_{t}-V_{0})$$

Molecular masses were estimated by making use of the inverse, linear correlation between *K*_av_ and the logarithm of the molecular mass [45].

### Enzyme kinetic analysis

Aldehyde dehydrogenase activity was measured at 30°C using a ThermoScientific Multiskan™ Microplate spectrophotometer. For Ald6p, reactions contained 100 mM sodium phosphate buffer (pH 7.3), containing 40 µM NADP^+^, 20 µM MgCl_2_ (Mg^2+^ has been previously identified as a cation activator of *S. cerevisiae* Ald6p and NADP^+^ as the enzyme’s preferred cofactor [47,48]) and varied concentrations of substrates ranging from 10–1200 µM. The long-chain (C_8_–C_13_) and phenolic aldehydes were dissolved in 1.7% (v/v) DMSO as a solvent carrier; this solvent has been previously shown to have little effect on the activity of aldehyde dehydrogenases [49,50]. Ald4p was assayed in the same buffer containing 20 µM KCl, 0.4 mM NAD^+^, 5 mM EDTA and 1 mM PMSF. K^+^ has been shown to act as a cation activator of *S. cerevisiae* Ald4p and this enzyme can function with either NAD^+^ or NADP^+^ as a cofactor [48,51].

Steady-state kinetic data was obtained in triplicates from the same 96-well plate with readings taken every 5 s. The initial, linear portion of the progress curve was identified by visual inspection and fitted by linear regression to give the initial rates (*v*) of change in absorbance at 340 nm. These rates were converted into molar units using the extinction coefficient of NADH (6.22 mM^−1^·cm^−1^) [52]. Rates of reactions were thus expressed as micromolar concentration of NAD(P)H formed per second.

The kinetic parameters (*V*_max_, *K*_0.5_ and Hill coefficient, *h*) were obtained by plotting the rates of reaction against substrate concentration and fitting the data to the equation below using non-linear regression as implemented in GRAPHPAD PRISM 6.0 (GraphPad Software Inc, CA, U.S.A.). All points were weighted equally.

$$v=V_{max}{\left[S\right]}^{h}/(K_{0.5}^{h}+{\left[S\right]}^{h})$$

Where, *V*_max_ is the maximum enzyme velocity, [S] is the concentration of substrate, *K*_0.5_ is the concentration of substrate that produces a half-maximal enzyme velocity (analogous to the Michaelis–Menten constant, *K*_m_, in non-cooperative enzymes) and *h* is the Hill coefficient [53,54].

### Differential scanning fluorimetry (DSF)

Enzyme aliquots were diluted in 50 mM HEPES, pH 7.3, to a concentration of 5–7 µM in a final volume of 20 µl. Sypro Orange (10×; manufacturer’s concentration definition) was mixed well and added. Determination of "melting temperatures" (T_m_) was done as previously described [46,55]. Cofactors (NADP^+^ or NAD^+^) and substrates were added as appropriate. Substrates were initially dissolved in 100% DMSO and diluted in buffer R (50mM HEPES–OH, pH 7.5, 150 mM NaCl, 10% (v/v) glycerol) as required. The concentration of DMSO never exceeded 1% (v/v).

## Results and discussion

### Sequence and predicted structure of Ald4p and Ald6p

Following PCR amplification of *S. cerevisiae var*. *boulardii ALD4* and *ALD6*, the coding sequences were determined and deposited in GenBank (accession numbers: KX022008 and KT869135 for *ALD4* and *ALD6* respectively). Alignment of the coding sequences using the *S. cerevisiae* S288c genome as reference revealed single nucleotide difference in the *S. cerevisiae var. boulardii ALD4* sequence (1342C>T) and five differences in the *S. cerevisiae var. boulardii ALD6* sequence (294C>T, 487A>G, 753A>G, 813G>A, 894T>C). Only the 487A>G substitution in *ALD6* altered the protein coding sequence, resulting in the replacement of the isoleucine at position 163 by a valine residue. No difference was observed in the protein coded for by *ALD4*. Multiple sequence alignment revealed an overall approximately 70% similarity (50% identity) between the human class 1 and 2 and both Ald4p and Ald6p (Supplementary Figure S1b), with conserved motifs equivalent of GXGXXG box at the Rossmann fold (residues ∼160–230) and residues at the catalytic channel (residues ∼200–410) (Supplementary Figure S1a). Although Ald4p is reported to be a mitochondrial enzyme in *S. cerevisiae*, it lacks a discernable targeting sequence (consensus: MLSLRQSIRFFKPATRTLCSSRYLL at the N-terminus [56]), as does the variant studied here.

**Figure 1:**
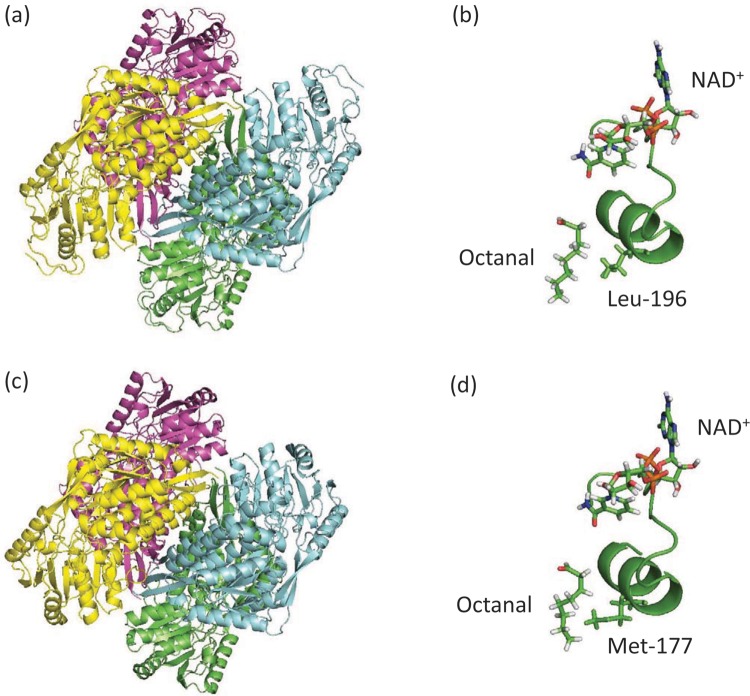
The predicted structure of *S. cerevisiae var. boulardii* Ald4p and Ald6p (**a**) The overall fold of Ald4p is shown as a tetramer, with each subunit in a different colour. (**b**) A close up of the active site showing the arrangement of the NAD^+^ cofactor and the aliphatic aldehyde substrate, octanal. Backbone corresponding to residues Pro^181^ to Ile^202^ is also shown. (**c**) The overall fold of Ald6p is shown as a tetramer, with each subunit in a different shade of grey. (**d**) A close up of the active site showing the arrangement of the cofactor and the aliphatic aldehyde substrate, octanal. Backbone corresponding to residues Pro^162^ to Ile^183^ is also shown. In (b) and (d), a key structurally equivalent residue in the active site is shown (Leu^196^ in Ald4p and Met^177^ in Ald6p). Note how the longer, bulkier methionine residue might sterically hinder non-aliphatic substrates.

Using the derived protein sequences of *S. cerevisiae var. boulardii* Ald4p and Ald6p, molecular models were built (Figure 1). As expected, these models show a high degree of similarity to other aldehyde dehydrogenase structures. For example, Ald4p has RMSD of 0.720 Å over 10088 equivalent atoms when compared with sheep liver aldehyde dehydrogenase (1BXS); Ald6p and this protein had an RMSD of 0.729 Å over 10476 equivalent atoms. Val^163^ in Ald6p (which differs from the previously reported sequences of *S. cerevisiae* Ald6p) does not form part of the active site and lies close to the oligomeric interface. It, therefore, seems unlikely that the conservative substitution of valine for isoleucine in our sequence compared with the *S. cerevisiae* S288c genome has any functional consequence for the enzyme.

Each subunit in Ald4p and Ald6p contains one active site and the residues forming each of these sites come from one subunit only. In our model, the cofactor and the substrate lie end-to-end in the active site with the nicotinamide ring of the cofactor orientated towards the aldehyde group of the substrate (in our models, the distance between the cofactor and the aldehyde group appears too large for efficient catalysis. We, therefore, assume that there will be some conformational changes in order to bring the two substrates closer together before the reaction can occur. This change is unlikely to affect the identity of the residues interacting with the substrates). While the cofactor is predicted to make many specific contacts with the enzyme, the aldehyde substrate appears to be located in a channel lined mainly with highly conserved hydrophobic residues as previously described in class 1, 2 and 3 human ALDHs [57–59]. Consequently, there are few specific contacts between the protein and this substrate leading us to hypothesize that both Ald4p and Ald6p can accommodate a range of different aldehyde substrates. This is consistent with previous observations on bacterial, fungal and higher eukaryotic aldehyde dehydrogenases [58–62]. We further noted that part of the aldehyde-binding site in Ald6p is formed by two methionine residues (Met^177^ and Met^178^). The structurally equivalent residues in Ald4p are Leu^196^ and Met^197^ (Figure 1b,d). We postulated that substitution of the less bulky, hydrophobic leucine residue at this position in the aldehyde-binding pocket might enable Ald4p to accommodate bulkier substrates such as cyclic, aromatic or branched chain aldehydes.

### Expression and purification of mitochondrial Ald4p, cytosolic Ald6p and its variant Ald6p.M177V

**Figure 2:**
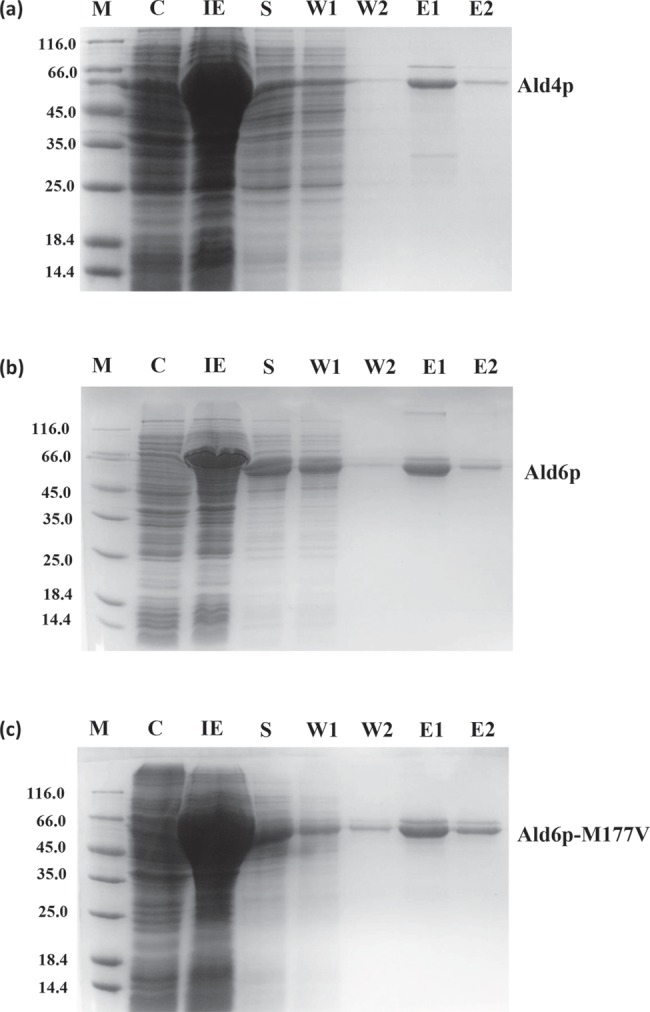
SDS/PAGE expression profiles and purification of (a) Ald4p (56.2 kDa), (b) Ald6p (54.4 kDa) and (c) Ald6p-M177V (54.4 kDa) using Rossetta™ (DE3) *E. coli* M: molecular mass marker 14.4–116.0 kDa (sizes shown to the left of the gel); C: cell extract; IE: induced expression; S: sonicate; W1, W2: affinity column wash; E1, E2: elutions (for details of buffers etc., see ‘Materials and methods’ section).

Ald4p and Ald6p (wild-type and variant Ald6p.M177V) could be expressed in, and purified from, *E. coli* cells (Figure 2). Typical yields of wild-type Ald6p and its variant were approximately 2 mg/l of bacterial cell culture (Figure 2b,c), but the yield of Ald4p was typically less than 1 mg/l culture (Figure 2a).

The resolution of the protein samples on SDS/PAGE (10% gels) showed small amounts of Ald6p at approximately four times the expected molecular mass (Figure 2b) suggesting the presence of some protein oligomers that are resistant to separation by SDS and heat treatment. Heat and SDS-resistant oligomerization have been observed in a number of other proteins [63,64]. The intensity of this band was not reduced by supplementation of the loading buffer with additional DTT (2 mM, results not shown).

### Both cytosolic and mitochondrial yeast aldehyde dehydrogenases are oligomers

**Figure 3:**
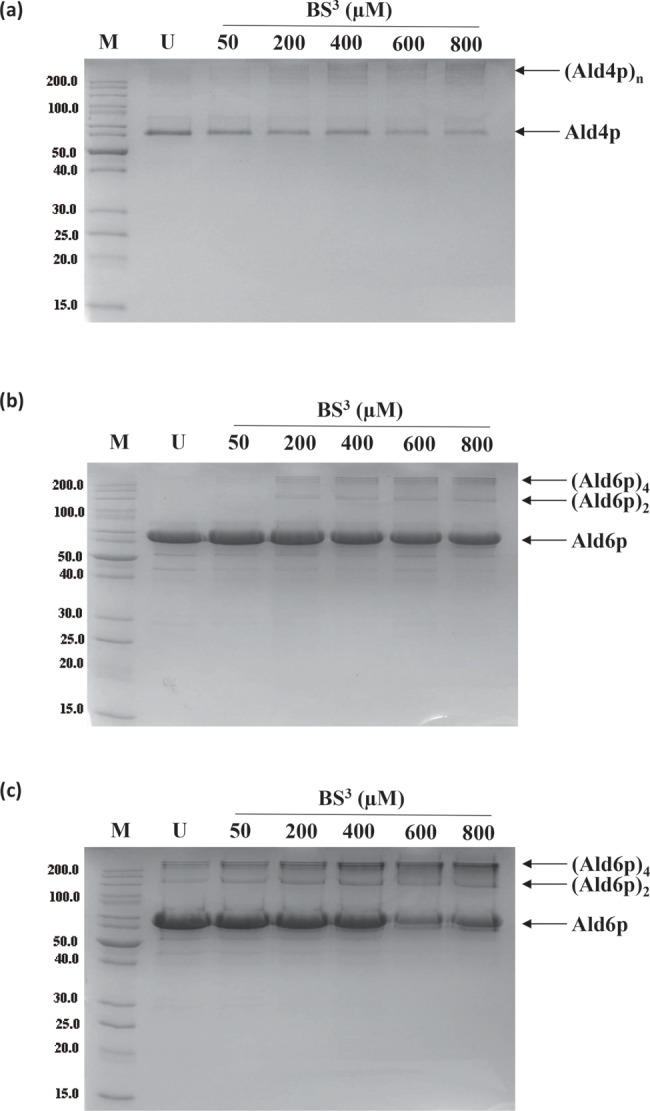
SDS/PAGE analysis of cross-linking of (a) Ald4p, (b) Ald6p and (c) Ald6p p.M177V with BS^3^ M: molecular mass marker 10.0–200.0 kDa (selected sizes shown to the left of the gel). U: untreated, purified ALDH; the remaining lanes show proteins treated with 50, 200, 400, 600 and 800 µM BS^3^ as indicated.

The ability of the three enzymes to form dimers and tetramers was further investigated using chemical cross-linking with BS^3^ (Figure 3). Resolution of the cross-linked products by SDS/PAGE (10% gel) revealed bands corresponding to an Ald6p dimer (∼110 kDa) and tetramer (∼220 kDa) (Figure 3b). However, the Ald6p.M177V variant showed oligomerization even in the absence of BS^3^ suggesting increased oligomeric stability compared with the wild-type protein. The intensity of these bands was greater following treatment with increasing concentrations of BS^3^ (Figure 3c). Oligomerization was also seen with Ald4p; however, neither discrete homodimer nor homotetramer forms were detected by SDS/PAGE (Figure 3a). Similar patterns of oligomerization was seen when cross-linking was done with 1–3% (v/v) glutaraldehyde (results not shown).

**Figure 4:**
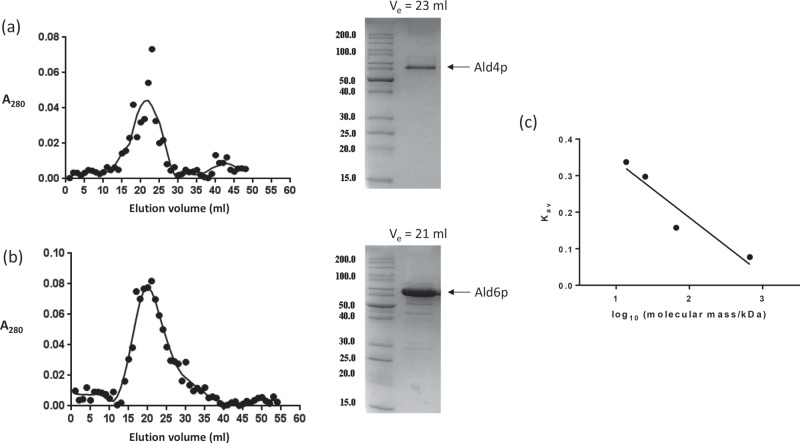
Analytical gel filtration elution profiles of (a) Ald4p (∼20 µM) and (b) Ald6p (∼60 µM) The points represent individual *A*_280_ readings and the line represents a trend line fitted by GraphPad Prism. The SDS profile shows the eluted fraction corresponding to the highest *A*_280_ read. (**c**) The calibration graph used for the estimation of molecular masses.

Oligomers were also detected by gel filtration chromatography for both Ald4p and Ald6p (Figure 4). The molecular mass of Ald6p was estimated to be approximately 220 kDa, which corresponds to a tetrameric assembly. The molecular mass of Ald4p, on the other hand was estimated to be approximately 151 kDa suggesting a trimeric arrangement. A non-tetrameric arrangement for an ALDH is unusual and our models of Ald4p show the protein as a tetramer. There is some, limited previous evidence for trimeric forms of *S. cerevisiae* aldehyde dehydrogenases [65]. These studies were based on crude extracts and are likely to represent a mixture of the isoenzymes now known to be present in this species. In the present study, cross-linked species corresponding to dimers, trimers and tetramers were detected. It is hard to imagine how the protein could exist as a trimer without substantial conformational changes, assuming that the basic fold of the monomer has been predicted correctly. Therefore, we assume that either the protein exists largely as a tetramer that runs anomalously in gel filtration chromatography or there is a dynamic equilibrium between a dimer and a ‘dimer of dimers’. Anomalies in gel filtration can arise from interactions between the protein and matrix, which retard the elution of the protein resulting in a lower estimated apparent molecular mass [66]. The previously detected trimers may represent intermediates in the formation of tetramers. Human ALDH3 is known to exist largely as a dimer due to an extended C-terminal tail that prevents tetramerization [67]. However, *S. cerevisiae* *var. boulardii* Ald4p lacks a C-terminal tail and, in our models, the shorter N-terminal extension did not appear to sterically hinder the formation of tetramers since it lies on the exterior of the predicted structure.

### Both Ald4p and Ald6p exhibit positive cooperativity, but the two enzymes show markedly different substrate specificity

Both Ald4p and Ald6p are active aldehyde dehydrogenases demonstrating positive cooperativity towards their aldehyde substrates (Table 1, Supplementary Figures S2, S3 and S4). This may be accounted for the stability of multi-subunit configuration of these enzymes where binding of the substrate to the first subunit increases its affinity for the subsequent subunits. Previously, positive cooperativity was reported in *S. cerevisiae* cytosolic aldehyde dehydrogenase for binding the cofactor NAD(P)^+^ following the substitution of an arginine residue with glutamate at position 480 [68]. Interestingly, no observation for cooperative kinetics with respect to the aldehyde substrates has previously been reported for yeast aldehyde dehydrogenases. The physiological significance of these cooperative kinetics with substrates is not yet known. In general, positively cooperative kinetics enable ‘switch-like’ responses to changes in substrate concentrations and are commonly seen in enzymes and pathways that need to be highly sensitive to small changes in substrate concentrations [69]. Ald6p showed activity with straight chain aliphatic aldehydes ranging from C_2_ to C_11_ and the branched chain, C_4_ aldehyde, isobutanal. Once the chain length extended beyond 11 carbon atoms, no activity was detected. However, it should be noted that, as the chain length increases, the aldehydes become less soluble in water and so the lack of activity may be partly explained by the failure of the compounds to dissolve rather than failure to interact with the enzyme’s active site. Wild-type Ald6p showed no activity with any of the cyclic or aromatic aldehydes tested. Interestingly, there was little correlation between activity and chain length, with the highest activity (judged by the ratio of *k*_cat_ to *K*_0.5_) being shown with propionaldehyde (Table 1). Like Ald6p, Ald4p was active with a range of aliphatic aldehydes, with hexanal being the ‘preferred’ substrate from those tested here. However, no activity could be detected with butyraldehyde (C_4_), not with any aldehydes with a chain length greater than nine carbon atoms. In contrast with Ald6p, this enzyme was active with benzaldehyde, although it lacked activity with the other cyclic and aromatic aldehydes tested (Table 1).

**Table 1:** Apparent steady-state kinetic parameters for *S. cerevisiae* Ald4p and Ald6p with a range of substrates

Substrate	Ald4p				Ald6p				Ald6p-pM177V			
	*k*_cat_ (s^−1^)	*K*_0.5_ (µM)	*k*_cat_/*K*_0.5_ (µM^−1^·s)	*h*	*k*_cat_ (s^−1^)	*K*_0.5_ (µM)	*k*_cat_/*K*_0.5_ (µM^−1^·s)	*h*	*k*_cat_ (s^−1^)	*K*_0.5_ (µM)	*k*_cat_/*K*_0.5_ (µM^−1^·s)	*h*
Acetaldehyde	(2.91 ± 0.12) × 10^−3^	20.32 ± 1.32	(1.43 ± 0.06) × 10^−4^	3.27 ± 0.71	(1.51 ± 0.03) × 10^−2^	205.3 ± 8.15	(7.38 ± 0.16) × 10^−5^	1.87 ± 0.11	(3.92 ± 0.62) × 10^−3^	49.13 ± 7.21	(7.98 ± 1.27) × 10^−5^	3.08 ± 0.81
Propionaldehyde	(2.91 ± 0.10) × 10^−3^	17.61 ± 1.16	(1.65 ± 0.06) × 10^−4^	2.76 ± 0.46	(1.44 ± 0.12) × 10^−2^	38.4 ± 5.14	(3.75 ± 0.33) × 10^−4^	1.63 ± 0.18	(1.44 ± 0.66) × 10^−2^	72.64 ± 37.41	(2.02 ± 0.92) × 10^−4^	1.86 ± 0.53
Isobutyraldehyde	(5.25 ± 2.30) × 10^−3^	81.71 ± 25.88	(6.43 ± 2.81) × 10^−5^	1.26 ± 0.28	(7.56 ± 0.36) × 10^−3^	91.00 ± 4.33	(9.01 ± 0.43) × 10^−5^	3.72 ± 0.56	(7.5 ± 0.78) × 10^−3^	37.00 ± 4.83	(2.02 ± 0.21) ×10^−4^	2.31 ± 0.44
Butyraldehyde	–*	–*	–*	–*	(3.22 ± 0.09) × 10^−2^	95.36 ± 2.79	(3.53 ± 0.10) × 10^−4^	4.12 ± 0.42	–*	–*	–*	–*
Valeraldehyde	(3.22 ± 0.16) × 10^−3^	30.21 ± 2.25	(1.06 ± 0.05) × 10^−4^	2.00 ± 0.21	(3.41 ± 0.15) × 10^−3^	21.32 ± 1.47	(1.6 ± 0.07) × 10^−4^	2.39 ± 0.38	(1.21 ± 0.07) × 10^−2^	37.17 ± 2.31	(3.26 ± 0.19) × 10^−4^	4.88 ± 1.00
Hexanaldehyde	(1.75 ± 0.05) × 10^−3^	12.43 ± 0.79	(1.41 ± 0.04) × 10^−4^	2.89 ± 0.51	(1.26 ± 0.38) × 10^−2^	49.19 ± 6.48	(1.98 ± 0.06) × 10^−4^	1.86 ± 0.51	(8.43 ± 0.58) × 10^−3^	19.88 ± 2.15	(4.24 ± 0.29) × 10^−4^	3.25 ± 1.14
Heptanaldehyde	(2.74 ± 0.13) × 10^−3^	23.80 ± 1.61	(1.15 ± 0.05) × 10^−4^	4.46 ± 1.34	(6.91 ± 2.24) × 10^−3^	53.81 ± 21.8	(9.65 ± 3.13) × 10^−5^	1.87 ± 0.58	(5.2 ± 0.18) × 10^−3^	16.99 ± 1.13	(3.06 ± 0.11) × 10^−4^	3.67 ± 0.77
Octanaldehyde	(2.32 ± 0.08) × 10^−3^	25.86 ± 1.16	(8.97 ± 0.31) × 10^−5^	4.00 ± 0.71	(1.46 ± 0.06) × 10^−3^	30.15 ± 6.67	(1.56 ± 0.07) × 10^−5^	3.6 ± 2.56	–*	–*	–*	–*
Nonanaldehyde	(2.52 ± 2.00) × 10^−3^	72.97 ± 6.51	(3.46 ± 2.74) × 10^−5^	1.86 ± 0.92	(1.74 ± 4.00) × 10^−2^	288.5 ± 0.744	(6.06 ± 13.8) × 10^−5^	1.24 ± 0.36	–*	–*	–*	–*
Decyl aldehyde	–*	–*	–*	–*	(3.27 ± 1.41) × 10^−3^	52.62 ± 32.29	(6.22 ± 2.69) × 10^−5^	1.61 ± 0.62	–*	–*	–*	–*
Undecyl aldehyde	–*	–*	–*	–*	(9.82 ± 2.80) × 10^−4^	49.08 ± 13.65	(2.00 ± 0.57) × 10^−5^	2.81 ± 1.20	–*	–*	–*	–*
Dodecyl aldehyde	–*	–*	–*	–*	–*	–*	–*	–*	–*	–*	–*	–*
Tridecyl aldehyde	–*	–*	–*	–*	–*	–*	–*	–*	–*	–*	–*	–*
Crotonaldehyde	–*	–*	–*	–*	–*	–*	–*	–*	–*	–*	–*	–*
Cyclohexane- carboxyaldehyde	–*	–*	–*	–*	–*	–*	–*	–*	(6.93 ± 0.3) × 10^−3^	26.97 ± 1.57	(2.57 ± 0.11) × 10^−4^	2.66 ± 0.37
Benzaldehyde	(9.16 ± 0.50) × 10^−4^	20.29 ± 1.04	(4.51 ± 0.25) × 10^−5^	7.83 ± 6.22^†^[Table-fn T1TFN2]	–*	–*	–*	–*	–*	–*	–*	–*
4-hydroxy- benzaldehyde	–*	–*	–*	–*	–*	–*	–*	–*	–*	–*	–*	–*
Vanillin	–*	–*	–*	–*	–*	–*	–*	–*	–*	–*	–*	–*
DL- glyceraldehyde	–*	–*	–*	–*	–*	–*	–*	–*	–*	–*	–*	–*

Activity of yeast Ald6p and Ald6p–M177V was assayed using 100 mM sodium phosphate buffer (pH 7.3) at 30°C containing 40 µM NADP^+^, 20 µM MgCl_2_ (Mg^2+^ being the cation activator) and varied concentrations of substrates. Long-chain (C_8_–C_13_) and aromatic aldehydes were dissolved in 1.7% (v/v) DMSO as a solvent carrier (see ‘Materials and methods’ section). Ald4p was assayed in the same reaction buffer containing 20 µM KCl (K^+^ acting as cation activator), 0.4 mM NAD^+^, 5 mM EDTA and 1 mM PMSF.

*No detectable activity.

^†^This error is very high. Fitting of *h* by non-linear methods can be challenging since large changes in this parameter can greatly affect the others. This value should be taken as evidence of positive cooperativity (which is also supported by the shape of the curve, Supplementary Figure S2) rather than a definitive estimate.

Inspection of the molecular model of the active sites of Ald4p and Ald6p revealed a possible reason for these differences in specificity. On one side of the Ald6p active site are two methionine residues (Met^177^ and Met^178^). These bulky side chains protrude into the active site filling some of the volume potentially sterically hindering the binding of a cyclic or aromatic substrate. In contrast, in Ald4p, the structurally equivalent residue was a leucine (Leu^196^). We, therefore, reasoned that replacement of Met^177^ residue with a smaller valine residue might enable Ald6p to interact with cyclic and/or aromatic aldehydes. Our hypothesis is supported by the sequence alignment with human aldehyde dehydrogenases (Supplementary Figure S1): both human ALDH1 and ALDH2, which are active with aliphatic and cyclic aldehydes [26], have smaller amino acid residues (valine and leucine respectively) at the position corresponding to Met^177^ in Ald6p. Furthermore, we have previously shown that an aldehyde dehydrogenase from *Candida dubliniensis* (which has an isoleucine at the equivalent position) is active with some cyclic and aromatic substrates [70].

The Ald6p.M177V variant is active with aliphatic aldehydes and showed an increased activity with medium chain- length aliphatic aldehydes (valeraldehyde and hexanaldehyde) compared with both wild-type Ald4p and Ald6p. However, its activity was only observed up to a chain length of seven carbons. Interestingly, it also gained activity with cyclohexanecarboxaldehyde, a substrate that the wild-type enzyme has no activity towards (Table 1). However, even with this change, there was no activity with benzaldehyde. This may be due to the lower reactivity of this aromatic aldehyde. This suggests that our hypothesis is partially correct and that the creation of additional volume at this point in the active site enables the binding and subsequent oxidation of bulkier aldehydes. However, the Ald6p.M177V has no activity towards benzaldehyde (unlike Ald4p) suggesting that there are additional determinants of substrate specificity in these enzymes. Ald6p.M177V, like Ald4p but not wild-type Ald6p, has no activity with butyraldehyde. This suggests that a valine or leucine at the position equivalent to 177 in Ald6p is incompatible with the binding of four carbon aldehydes. It is hard to provide a definitive explanation in the absence of an experimentally determined structure. However, it may be that shorter aldehydes (with three or fewer carbons and isobutaraldehyde which is branched) bind without significant interaction with this residue. The branched nature of valine or leucine may create some steric hindrance to four carbon aldehydes, which can be overcome by the greater binding energy expected in substrates with longer carbon chains.

### Substrates and cofactors affect the thermal stability of yeast aldehyde dehydrogenases

The Ald6p-M177V variant is more thermally stable than the wild-type with a melting temperature ∼4°C higher (Table 2, Supplementary Figure S5). This demonstrates that the M177V substitution increases the stability of the protein. Given that it was easier to detect oligomers in the variant (see above, Figure 3c), this most likely results from tighter binding between the subunits of the oligomers. Ald4p has similar thermal stability to wild-type Ald6p (Table 2). NAD(P)^+^ binding caused a significant (*P*<0.05) increase in the *T*_m_ for Ald4p and wild-type Ald6p demonstrating that this increases the overall stability towards heat denaturation of all three proteins (Table 2, Supplementary Figure S5). Addition of the aldehyde substrates (in the presence of the appropriate cofactor) generally increased the thermal stability of Ald4p, but most had little or no effect on Ald6p (Table 2, Supplementary Figure S5). Aldehyde dehydrogenases have an ordered, ternary complex mechanism in which NAD(P)^+^ binds first. These data suggest that cofactor binding results in a small stabilization of the enzyme, perhaps due to a reorganization of the structure which then permits aldehyde binding.

**Table 2:** Thermal stability (*T*_m_) of *S. cerevisiae var. boulardii* Ald4p and Ald6p (WT and p.M177V variant)

Substrate or cofactor	*T*_m_ (°C)		
	Ald4p	Ald6p	Ald6p–M177V
Untreated	62.5 ± 0.0	62.5 ± 0.5	66.5 ± 0.2
NAD^+^	63.1 ± 0.1*	nd	nd
NADP^+^	nd	63.9 ± 0.2*	63.5 ± 0.0*
Propionaldehyde	65.0 ± 0.2*	63.2 ± 0.7	66.7 ± 0.0
Valeraldehyde	65.5 ± 0.0*	nd	67.0 ± 0.5
Octanal	nd	62.7 ± 0.6	66.7 ± 0.6
Decanal	64.5 ± 0.5*	62.3 ± 0.3	65.8 ± 0.4*
Tridecanal	nd	61.8 ± 0.0	66.0 ± 0.5
Crotonaldehyde	65.5 ± 0.1*	63.0 ± 0.5	67.0 ± 0.5
Cyclohexanecarboxaldehyde	63.5 ± 0.2*	61.2 ± 0.2*	65.7 ± 0.2*
Benzaldehyde	63.5 ± 0.2*	61.0 ± 0.5*	66.0 ± 0.0
4-hydroxybenzaldehyde	64.5 ± 0.3*	62.3 ± 0.5	66.5 ± 0.1*
Vanillin	64.5 ± 0.4*	62.5 ± 0.5	66.2 ± 0.5

Aldehyde substrates were measured with 2 mM substrate and cofactor (1.5 mM NAD^+^ for Ald4p, 2 mM NADP^+^ for Ald6p). Cofactors were measured with 1.5 mM NAD^+^ for Ald4p and 2.0 mM NADP^+^ for Ald6p.

nd, not determined.

*Indicates a statistically significant difference (*P*<0.05). Experiments with cofactors were compared with the appropriate untreated enzyme and experiments with cofactor and aldehyde were compared with the appropriate one with cofactor only.

## Conclusions

Both mitochondrial Ald4p and cytosolic Ald6p were observed to be prevalent in oligomeric configurations. Our study also showed cooperativity of these enzymes towards aldehyde substrates, a phenomenon commonly observed in oligomeric enzymes. Based on the molecular models, we hypothesized that the difference in specificity for aliphatic and aromatic aldehydes was partly due to a bulky methionine residue partially sterically hindering the binding of cyclic or aromatic substrates. Substitution of Met^177^ altered the specificity of Ald6p, suggesting that other alterations to the sizes and properties of amino acids in this region of the protein may enable further modification of the substrate range of aldehyde dehydrogenases.

## Abbreviations

ALDH: Aldehyde dehydrogenase
BS^3^: bis(sulfosuccinimidyl) suberate
DSF: differential scanning fluorimetry
PDB: Protein Data Bank
